# Anti-Sweep Jamming Design and Implementation Using Multi-Channel Harmonic Timing Sequence Detection for Short-Range FMCW Proximity Sensors

**DOI:** 10.3390/s17092042

**Published:** 2017-09-06

**Authors:** Zhijie Kong, Ping Li, Xiaopeng Yan, Xinhong Hao

**Affiliations:** School of Mechatronical Engineering, Beijing Institute of Technology, Beijing 100081, China; ethankong@163.com (Z.K.); liping85@bit.edu.cn (P.L.); yanxiaopeng@bit.edu.cn (X.Y.)

**Keywords:** FMCW, proximity sensor, anti-sweep jamming, timing sequence detection, ranging

## Abstract

Currently, frequency-modulated continuous-wave (FMCW) proximity sensors are widely used. However, they suffer from a serious sweep jamming problem, which significantly reduces the ranging performance. To improve its anti-jamming capability, this paper analyzed the response mechanism of a proximity sensor with the existence of real target echo signals and sweep jamming, respectively. Then, a multi-channel harmonic timing sequence detection method, using the spectrum components’ distribution difference between the real echo signals and sweep jamming, is proposed. Moreover, a novel fast Fourier transform (FFT)-based implementation was employed to extract multi-channel harmonic information. Compared with the traditional band-pass filter (BPF) implementation, this novel realization scheme only computes FFT once, in each transmission cycle, which significantly reduced hardware resource consumption and improved the real-time performance of the proximity sensors. The proposed method was implemented and proved to be feasible through the numerical simulations and prototype experiments. The results showed that the proximity sensor utilizing the proposed method had better anti-sweep jamming capability and ranging performance.

## 1. Introduction

Frequency-modulated continuous wave (FMCW) sensors constantly transmit and receive signals and is thus capable of maintaining a high signal-to-noise ratio with much less peak power than a corresponding pulse sensor system. Due to its simple structure, the FMCW sensors are widely used in a plurality of areas [1], such as healthcare [2], security [3], navigation [4], vehicle collision warning system [5], geodesy [6], helicopter and aircraft landing control [7], and other related areas. The ranging performance is a key parameter for the FMCW sensor. However, it is significantly affected by unintended interference [8] and intended jamming [9]. Among them, the intended jamming has become a serious threat to FMCW sensors, especially sweep jamming [10,11], which combines the properties of both spot jamming and barrage jamming by rapidly sweeping a narrow band of jamming signals over a wide frequency band [12]. Hence, many previous studies have focused on increasing the anti-sweep jamming capability of short-range proximity sensors.

Prior studies to increase the anti-sweep jamming capability of FMCW sensors can be categorized into two categories: transmitted waveform design [5,13,14,15] and beat signal processing [16,17,18,19]. Reference [5] designed a new transceiver and utilized the fractional Fourier transform (FRFT), which was sensitive to the FM slope, to suppress jamming. Perrin used the spectrum characteristics of echoes to detect targets [16], while Xiao et al. [17] distinguished the target position according to the Doppler characteristics of different encounters process. Based on an analysis of difference between the target echoes and jamming signal, Huang et al. [18] and Li et al. [19] utilized machine learning to classify the target and jammer, respectively. 

While various methods have been investigated to improve the anti-jamming capacity of FMCW sensors, it is of great importance to have a specific method for short-range FMCW proximity sensors. The special operating environment leads to two issues that generally do not exist in conventional radar designs. Due to the signal leakage, close range, and the generally narrow antenna beam, the target is only partially illuminated, leading to a relatively low signal-to-noise rate (SNR) [20,21]. Due to the small volume and power limitations, the hardware is generally simple and cost-effective, resulting in implementation difficulties of the complex circuits and algorithms.

One kind of proximity sensors uses harmonic signal information to detect targets. Through analyzing the spectrum of a short-range FMCW proximity sensor’s beat signal, it was found that the spectral harmonic component peaked at some specific distances. Existing sensors usually use BPF to extract the harmonic signal. However, the filter design has a direct effect on the suppression of jamming [22,23,24]. For instance, when more harmonic signals are used, the BPF-based harmonic acquisition method consumes a larger number of system resources, causing a non-negligible processing delay and performance decrease.

This paper presents an anti-sweep jamming design and its implementation for short-range FMCW proximity sensors using the multi-channel harmonic timing sequence detection method. The harmonic peak amplitude information and timing information of multi-channels are used to suppress the jamming. To reduce the resource consumption, our study implements the harmonic timing detection based on FFT. The multi-channel harmonic envelope information is acquired using FFT once, in each transmission cycle. The main contributions of this paper are as follows: (1) the failure mechanism of the sensor under sweep jamming is analyzed; (2) an anti-jamming method of multi-channel harmonic timing detection is proposed and implemented based on FFT; and (3) the anti-jamming capability is improved and resource usage is reduced.

The rest of the paper is organized as follows: the principles of short-range FMCW proximity sensors, the construction of the sweep jamming model, and the analysis of the theoretical basis of anti-sweep jamming are introduced in Section 2. Section 3 demonstrates the implementation of multi-channel harmonic timing detection based on the BPF and FFT, respectively. The simulation analysis of two harmonic timing detection methods is presented, and the sensor prototype is designed to verify the ranging and anti-jamming capability in Section 4, which is followed by our conclusions in Section 5.

## 2. Failure Mechanism Analysis of Short-Range FMCW Proximity Sensor under Sweep Jamming 

### 2.1. FMCW Harmonic Ranging Principle

**Figure 1:**
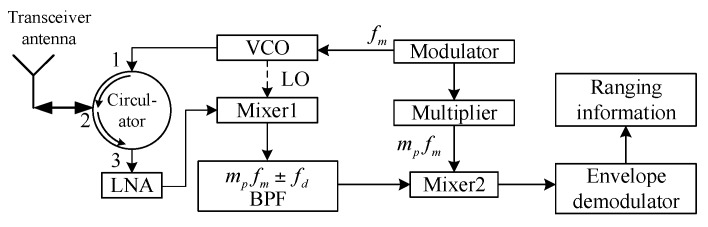
Block diagram of a short-range FMCW proximity sensor (LNA: low-noise amplifier; VCO: voltage-controlled oscillator; LO: local signal).

The FMCW sensor, shown schematically in Figure 1, transmits an FM signal, and mixes the transmitted signal and the target echo signal to obtain the beat signal after low-pass filtering. From the beat signal, the range information can be calculated.

The signal from the voltage-controlled oscillator (VCO) (controlled by the modulator) enters Port 1 of the circulator. Almost all of the energy of this signal passes from Port 2 to the transceiver antenna and is radiated toward the target. The expression of the transmission modulated by triangular waveform is

(1) $$s_{t}(t)=\left\{ \begin{array}{ll} A_{t}\cos\left\{2\pi [f_{c}+(4n+1)\Delta F]t-\pi \beta t^{2}\right\} & nT\leq t\leq nT+\frac{T}{2}\\ A_{t}\cos\left\{2\pi [f_{c}-(4n+3)\Delta F]t+\pi \beta t^{2}\right\} & nT+\frac{T}{2}\leq t\leq (n+1)T \end{array}\right.$$

where $A_{t}$ is the amplitude of the transmitted signal, $f_{c}$ is the carrier frequency, ΔF is half of bandwidth, β is the FM slope, n is the number of transmission, T is the period of modulation signal, and $f_{m}=\frac{1}{T}$ is the frequency of the modulation signal. To ensure the achievable implementation and maximum range detection, $f_{m}$ is set to be $f_{d}\ll f_{m}\ll \frac{c}{4R_{\max}}$, where $f_{d}$ is the Doppler signal produced by relative motion, c is the speed of light, and $R_{\max}$ is the maximum detection range of the proximity sensors.

The signal reflected is an attenuated and delayed version of the transmitted signal, expressed as $s_{r}(t)=A_{e}\cdot s_{t}(t-\tau )$, where $A_{e}$ is the attenuation factor, and τ is the round-trip time for the signal to propagate from the sensor system and back. The echo signal enters Port 2 of the circulator, passes to Port 3 and on to mixer1. Another port of mixer1 is connected to the local signal $s_{l}(t)$ with amplitude $A_{l}$. The output of mixer1 (called the beat signal) can be expressed as

(2) $$s_{b}(t)=\left\{ \begin{array}{l} \frac{A_{e}A_{t}A_{l}}{2}\cos\{2\pi \beta t^{2}-2\pi \beta \tau t-16\pi nt-2\pi [f_{c}-(4n-1)\Delta F]\tau +\pi \beta \tau^{2}\},\;nT\leq t<nT+\tau \\ \frac{A_{e}A_{t}A_{l}}{2}\cos\{2\pi \beta \tau t-2\pi [f_{c}+(4n+1)\Delta F]\tau -\pi \beta \tau^{2}\},\;nT+\tau \leq t<nT+\frac{T}{2}\\ \frac{A_{e}A_{t}A_{l}}{2}\cos\{2\pi \beta t^{2}-2\pi \beta \tau t-2\pi (8n+4)t+2\pi [f_{c}+(4n+1)\Delta F]\tau +\pi \beta \tau^{2}\},\;nT+\frac{T}{2}\leq t<nT+\frac{T}{2}+\tau \\ \frac{A_{e}A_{t}A_{l}}{2}\cos\{2\pi \beta \tau t+2\pi [f_{c}-(4n+3)\Delta F]\tau -\pi \beta \tau^{2}\},\;nT+\frac{T}{2}+\tau \leq t<(n+1)T \end{array}\right.$$

As τ≪T for the proximity sensor [22], the beat signal between nT≤t<nT+τ and $nT+\frac{T}{2}\leq t<nT+\frac{T}{2}+\tau$ can be ignored. The spectrum of $s_{b}(t)$ is

(3) $$F_{b}(f,\tau )=\frac{A_{e}A_{t}A_{l}}{2}\;\sum k(m,\tau )\;\delta (f-mf_{m}\pm f_{d})$$

where m is the harmonic number, and

(4) $$k(m,\tau )=\left\{ \begin{array}{l} (1-\frac{2\tau }{T})|(\sin c[(2\pi \beta \tau -2\pi mf_{m})(\frac{T}{4}-\frac{\tau }{2})]+\sin c[(2\pi \beta \tau +2\pi mf_{m})(\frac{T}{4}-\frac{\tau }{2})])\cos(2\pi f_{c}\tau )|,m\;\mathrm{is}\;\mathrm{even}\\ (1-\frac{2\tau }{T})|(\sin c[(2\pi \beta \tau -2\pi mf_{m})(\frac{T}{4}-\frac{\tau }{2})]-\sin c[(2\pi \beta \tau +2\pi mf_{m})(\frac{T}{4}-\frac{\tau }{2})])\sin(2\pi f_{c}\tau )|,m\;\mathrm{is}\;\mathrm{odd} \end{array}\right.$$

As shown in Equation (3), the spectrum of the beat signal consists of $mf_{m}\pm f_{d}$. The energy of the separate spectrum is described in Equation (4), which is composed of two Sinc function. The k(m,τ) display peaks when $(2\pi \beta \tau -2\pi mf_{m})(\frac{T}{4}-\frac{\tau }{2})=0$, deriving

(5) m=8ΔF⋅Rc

As shown above, when the predetermined distance $R_{p}$ is given, the corresponding harmonic number $m_{p}$ can be obtained from Equation (5). Hence, in practice, the detection of predetermined distance $R_{p}$ is always converted to the detection of the signal peak of $m_{p}$ harmonic. Generally, $m_{p}$ is designed to be an even number to suppress the damage of the leakage signal [25]. Equation (5) indicates that, when the sensor detects the target and the distance is reduced, the high-to-low-order harmonic signal peaks appear in turn and that the range resolution is $\Delta R=\frac{c}{4\Delta F}$.

The harmonic signal carrying Doppler information is obtained by BPF. The output of BPF is

(6) $$s_{bp}(t)=\frac{A_{e}A_{t}A_{l}}{2}\;k(m_{p},\tau )\;\cos[2\pi (m_{p}f_{m}\pm f_{d})t]$$

The $s_{bp}(t)$ is mixed with the reference signal in mixer2. The frequency of reference signal is $m_{p}f_{m}$. The output of mixer2 is passed to the envelope demodulator (shown in Figure 1), and the obtained Doppler signal is expressed in Equation (7). The threshold detection is performed using the Doppler signal to provide the ranging information.

(7) $$s_{d}(t)=\frac{A_{e}A_{t}A_{l}}{2}\;k(m_{p},\tau )\;\cos(2\pi f_{d}t)$$

### 2.2. Sweep Jamming Strategy

In sweep jamming, the jammer sweeps its frequency from one to the other and does not share its power among multiple frequencies.

The parameters of sweep jamming are as follows: the start frequency $f_{j0}$, the stop frequency $f_{jN}$, the frequency step $\Delta f_{j}$, the dwell time $T_{dw}$, and the sweep number N=(fjN−fj0)Δfj. The instantaneous frequency of the sweep jamming signal is

(8) $$f_{j}(t)=f_{j0}+\Delta f_{j}\cdot \sum_{k=0}^{N-1}k\cdot P_{T_{dw}}(t-\frac{T_{dw}}{2}-kT_{dw})$$

where k=0,1,⋯,N−1, and PTdw(t)={1,|t|≤Tdw20,others.

The phase of sweep jamming is

(9) $$\varphi_{j}(t)=2\pi \int_{0}^{t}f_{j}(t)dt=2\pi \left[(f_{j0}+k\cdot \Delta f_{j})\cdot t-\frac{k\cdot (k+1)}{2}\Delta f_{j}\cdot T_{dw}\right]$$

where $k\cdot T_{dw}<t\leq (k+1)\cdot T_{dw},\;k=0,1,\cdots ,N-1$.

**Figure 2:**
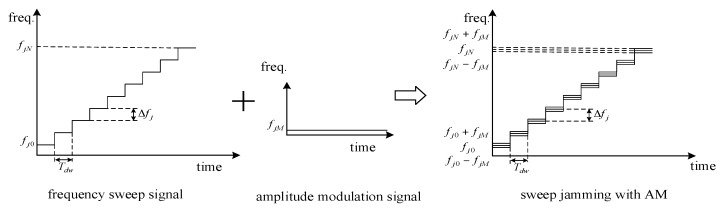
Block diagram of time–frequency analysis for the sweep jamming signal with AM.

Sweep jamming is generally used with amplitude modulation (AM), e.g., cos wave amplitude modulation, so the jamming signal $s_{j}(t)$ can be expressed as

(10) $$s_{j}(t)=[A_{j}+A_{jM}\cos(2\pi f_{jM}t+\varphi_{jM})]\cos(\varphi_{j}(t))$$

where $A_{j}$ is the amplitude of carrier signal, $A_{jM}$ is the amplitude of AM signal, $f_{jM}$ is the frequency of AM signal, and $\varphi_{jM}$ is the initial phase of the AM signal, assuming $\varphi_{jM}=0$ here. The amplitude modulation results in the spectrum shifting of $f_{j}(t)$, e.g., at the kth sweep point, and the instantaneous frequency consists of both $f_{j0}+k\cdot \Delta f_{j}\pm f_{jM}$ and $f_{j0}+k\cdot \Delta f_{j}$ after AM, as shown in Figure 2.

### 2.3. Failure Mechanism Analysis

The sweep jamming signal is received by the sensor transceiver and mixed with the LO; the beat signal under jamming can be expressed as

(11) $$\begin{array}{cc} s_{ij}(t) & =\frac{A_{l}}{2}[A_{j}+A_{jM}\cos(2\pi f_{jM}t)]\cdot \cos[\varphi_{j}(t)-\varphi_{l}(t)]\\ & =s_{ij1}(t)\cdot s_{ij2}(t) \end{array}$$

where $\varphi_{L}(t)$ is the phase of local signal, and

(12) $$\begin{array}{l} s_{ij1}(t)=\frac{A_{l}}{2}[A_{j}+A_{jM}\cos(2\pi f_{jM}t)]\\ s_{ij2}(t)=\cos[\varphi_{j}(t)-\varphi_{l}(t)] \end{array}$$

The Fourier transform (FT) of $s_{ij}(t)$ is $S_{ij}(f)$:

(13) $$S_{ij}(f)=S_{ij1}(f)*S_{ij2}(f)$$

where $S_{ij1}(f)$ and $S_{ij2}(f)$ are the FT of $s_{ij1}(t)$ and $s_{ij2}(t)$, respectively. The first item on the right side of Equation (13) shifts the spectrum of $S_{ij2}(f)$ and simulates the Doppler phenomenon during the encounter. The spectrum of $s_{ij}(t)$ is mainly dependent on $s_{ij2}(t)$.

(14) $$s_{ij2}(t)=\left\{ \begin{array}{l} \cos\left\{\begin{array}{l} 2\pi [(f_{c}+(4n+1)\Delta F-f_{j0}-k\cdot \Delta f_{j})t\\ -\pi \beta t^{2}+\frac{k\cdot (k+1)}{2}\Delta f_{j}\cdot T_{dw}] \end{array}\right\},\;k\cdot T_{dw}<nT\leq t\leq nT+\frac{T}{2}\leq (k+1)\cdot T_{dw}\\ \cos\left\{\begin{array}{l} 2\pi [(f_{c}-(4n+3)\Delta F-f_{j0}-k\cdot \Delta f_{j})t\\ +\pi \beta t^{2}+\frac{k\cdot (k+1)}{2}\Delta f_{j}\cdot T_{dw}] \end{array}\right\},k\cdot T_{dw}<nT+\frac{T}{2}\leq t\leq (n+1)T\leq (k+1)\cdot T_{dw} \end{array}\right.$$

The dwell time of sweep jamming is in the order of milliseconds, while the FM period is in the order of microseconds. Consequently, the sweep point is considered to be the same in a transceiver cycle. $s_{ij2}(t)$ can then be expressed as

(15) $$s_{ij2}(t)=\cos[2\pi (f_{c}-f_{j0}-k\cdot \Delta f_{j})t]\sum_{m=-\infty }^{+\infty }a_{m}e^{j2\pi mf_{m}t}$$

(16) $$a_{m}=\cos[\frac{\pi {\left(\mu +m\right)}^{2}}{4\mu }][C(a)+C(b)]-\sin[\frac{\pi {\left(\mu +m\right)}^{2}}{4\mu }][S(a)+S(b)]$$

where C(a), C(b), S(a), and S(b) are Fresnel functions, and $\mu =\frac{\Delta F}{f_{m}},a=\frac{\mu -m}{\sqrt{2\mu }},b=\frac{\mu +m}{\sqrt{2\mu }}$. The spectrum of $s_{ij}(t)$ is composed of $f_{c}-f_{j0}-k\cdot \Delta f_{j}\pm mf_{m}\pm f_{jM}$, with bandwidth 2ΔF.

The center frequency of sweep jamming is generally equal to the carrier frequency, and sweep bandwidth covers the bandwidth of the sensor. At the beginning of the sweep, the non-cooperative relationship between the sweep jamming and sensor transmission signal keeps the effective jamming signal out of the BPF’s passband; hence, the sensor is not jammed. With changes in the sweep frequency step, the effective jamming signal gradually passes through the BPF’s passband, that is, the signal appears in $m_{p}f_{m}\pm f_{jM}$. While the jamming signal’s energy is large enough, the harmonic energy satisfies the condition for ranging, causing the sensor to be jammed. 

When the sensor receives the sweep jamming signal, the corresponding peaks appear randomly in a certain number of harmonics, determined by the jamming parameters. However, when the sensor detects the target and its distance is reduced, the high-to-low-order harmonic signal peaks appear in a regular sequence. Therefore, harmonic peak timing sequence detection can be used to differentiate between the target echo and jamming signal.

## 3. Design and Implementation of Multi-Channel Harmonic Timing Sequence Detection

### 3.1. Multi-Channel Harmonic Timing Sequence Detection Based on BPF

**Figure 3:**
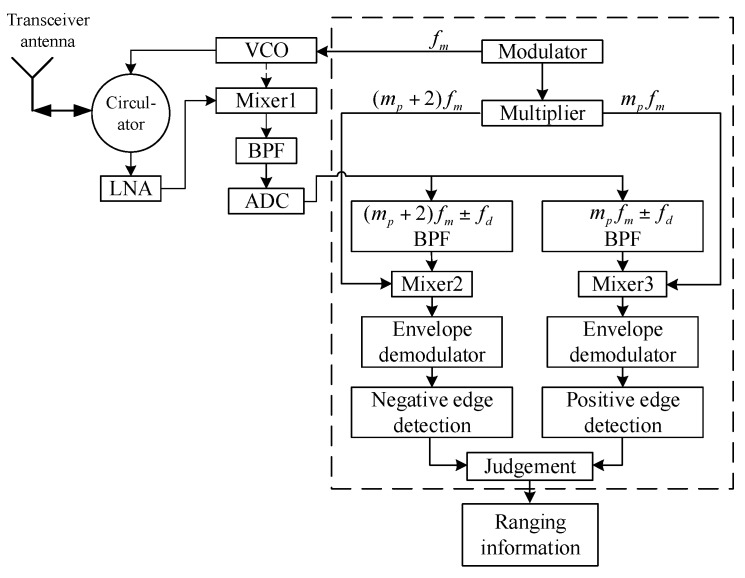
Block diagram of multi-harmonic timing sequence detection based on BPF.

The block diagram of the designed method based on BPF is shown in Figure 3.

Unlike the scheme in Figure 1, the newly designed sensor comprehensively combined dual-channel information to acquire the predetermined distance $R_{p}$. The negative edge of the $(m_{p}+2)f_{m}\pm f_{d}$ channel and the positive edge of $m_{p}f_{m}\pm f_{d}$ channel were detected simultaneously. Only when the negative edge comes earlier than the positive edge is the ranging condition satisfied.

Under the condition of sweep jamming, the spectrum of the beat signal is composed of $f_{c}-f_{j0}-k\cdot \Delta f_{j}\pm mf_{m}\pm f_{jM}$, the valid signal can pass through a dual-channel passband, but the amplitudes of both channels appear randomly during the dwell time of sweep jamming; when the point of sweep frequency comes, the positive edge and negative edge appear simultaneously, resulting in a mismatch of harmonic timing sequence detection.

More channels mean more obvious difference in timing sequence among them, which provides a more rigorous judging condition and better anti-jamming capability. However, this causes an implementation problem at the same time. Regardless of whether the analog filter group or digital filter is utilized, extra system resource consumption will be inevitable when more channels are employed, even though some signal processors such as the Field-Programmable Gate Array (FPGA) provide a special IP core. A larger number of system resources are consumed when the filters’ orders are relatively high, causing a non-negligible processing delay and performance decrease. To overcome this disadvantage, the anti-sweep jamming sensor based on FFT was proposed and designed.

### 3.2. Multi-Channel Harmonic Timing Sequence Detection Based on FFT

**Figure 4:**
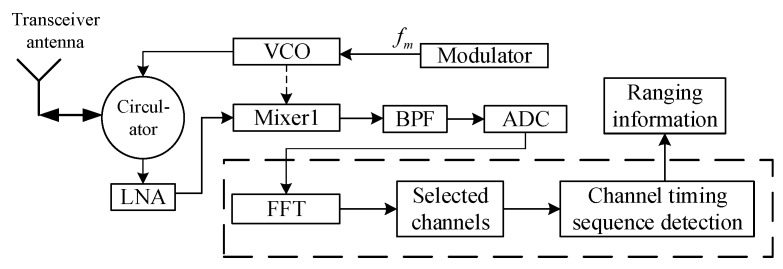
Block diagram of multi-harmonic timing sequence detection based on FFT (The operations in the dashed box are implemented using a digital device).

The block diagram of the designed sensor based on FFT is shown in Figure 4.

**Figure 5:**
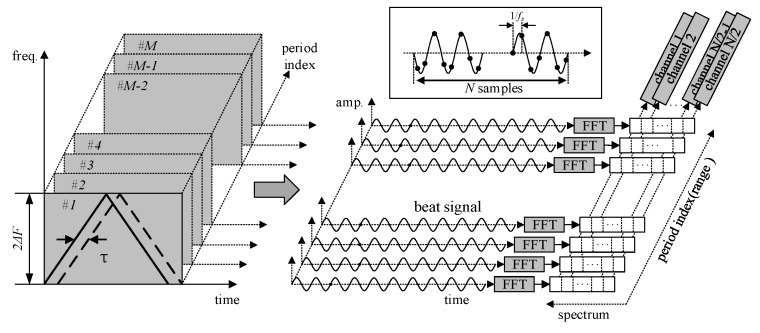
Basic concept of FFT-based harmonic timing sequence detection.

Different from the FFT-based instantaneous frequency estimation sensor, the designed sensor, instead of using instantaneous frequency, uses separate components of the beat signal spectrum to achieve harmonic timing sequence detection. The beat signal is sampled by the analog-to-digital converter (ADC) and then transformed using the FFT in the digital signal processor, and the multi-channel signal (changing with range) can be obtained as shown in Figure 5. When the amplitude conditions and timing sequence detection are met, the processor outputs the ranging information.

For the anti-sweep jamming sensor based on FFT, FFT is performed once during each transmission cycle to obtain the multi-channel harmonic amplitude. Furthermore, as computational complexity is related to the number of FFT points, the amount of digital resources can be significantly reduced by using this implementation method.

## 4. Simulated and Measured Results Discussion

We used numerical simulations and experimental tests to verify the key performances of the designed sensor: reliable ranging performance and improved anti-sweep jamming performance. In the first scenario, to test the ranging performance, only the target signal existed during the simulation. In the second scenario, the hardware resource usage of the proposed methods (based on the digital BPF implementation and the FFT implementation) were studied. In the third scenario, the outputs of the multi-channels were obtained under sweep jamming by the simulations and prototype experiments. The proposed multi-channel harmonic timing sequence detection was used to range. After numerous Monte Carlo simulations and experimental tests, the corresponding anti-jamming success rates were collected to quantitate the anti-sweep jamming performance.

### 4.1. Ranging Performance Simulation and Analysis

The ranging performance of the BPF and FFT implementation scheme (based on the timing sequence detection method) were simulated when the proximity sensor approached the target. The simulation parameters are illustrated in Table 1.

**Figure 6:**
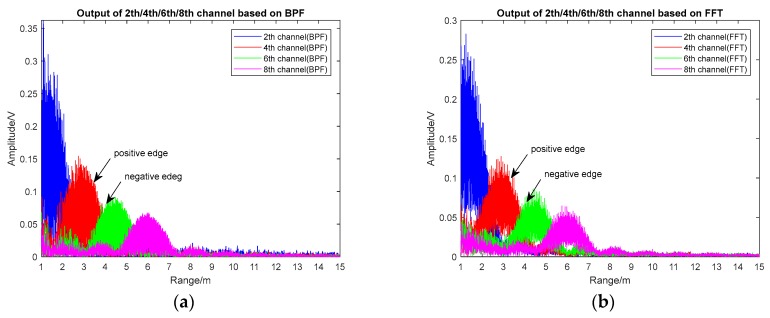
The outputs of the 2th/4th/6th/8th harmonic channels (SNR = −10 dB). (**a**) The BPF-based method, and (**b**) the FFT-based method.

**Figure 7:**
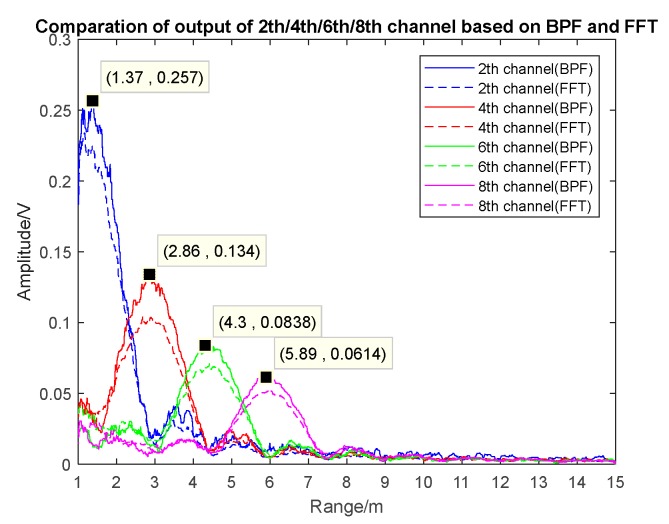
A comparison of the 2th/4th/6th/8th harmonic channels based on the BPF and FFT scheme (SNR = −10 dB).

As the proximity sensor works on the condition of low SNR, the background noise of additive white Gaussian noise was added in the simulation and the SNR was −10 dB. The outputs of the 2th/4th/6th/8th harmonic channels of the BPF-based and FFT-based realization scheme are shown in Figure 6a,b and Figure 7.

As shown in Figure 6, when the sensor gradually approached the target, the envelope peak of the 8th/6th/4th/2th harmonic signals appeared successively, and the envelope peak of different harmonic signals increased; namely, the peak of the 2nd harmonic was higher than that of the other three channels. Between the two adjacent signal peaks, the high-order harmonic signal was on the negative edge and the low-order harmonic signal was on the positive edge; thus, harmonic timing detection was achieved when a negative edge was followed by a positive edge.

Figure 7 demonstrates that the harmonic envelope based on FFT was basically consistent with the one based on BPF, and the peak points were close to the theoretical points (theoretical peak locations: 1.5 m, 3 m, 4.5 m, and 6 m, respectively). The maximum ranging error was 0.2 m, which was caused by the increase in echo power when the distance decreased. The range resolution of both methods was 1.5 m, which matched the theoretical value. In the premise of obtained multi-channel harmonic envelopes, timing sequence detection could be achieved through detecting the positive and negative edges.

### 4.2. Implementation Complexity Analysis

**Figure 8:**
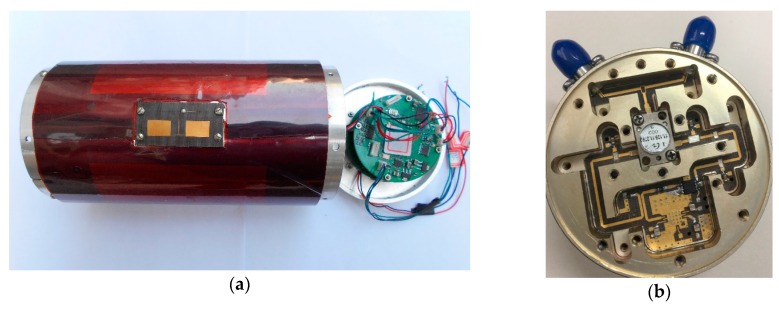
The designed anti-jamming sensor prototype. (**a**) The transceiver antenna and signal processing module, and (**b**) the RF module.

The short-range FMCW proximity sensor prototype was designed based on the structure described in Figure 4. Images of the prototype and the RF module are provided in Figure 8a,b.

The sub-parts of the RF module were designed using the sub-cavity to reduce the RF signal leakage, and signal processing was completed using the Field-Programmable Gate Array (FPGA). BPF- and FFT-based harmonic timing detection using three channels were implemented on the same hardware, and a comparison of the resources used is shown in Table 2.

The implementation of an anti-sweep jamming sensor based on FFT reduced the resource usage significantly; in particular, the number of multipliers used was about 38% of the number that the BPF-based sensor used. When using the FPGA for FFT with the system clock at 50 MHz, and 128 points, FFT takes time at the μs level, which satisfies the proximity sensor real-time requirements. To further reduce resource consumption, a harmonic signals envelope could be extracted using data collected on a single up-slope or down-slope, which has been verified.

The maximum detection range was considered in the design of the RF and signal processing modules. The output power of the transmitted signal was 13 dBm and the transceiver antenna gain was 4 dB. The echo was passed to LNA to ensure that the output of mixer1 was in the order of the mV level. A 12-bit ADC (resolution 1000/4096 mV) was used to sample the beat signal. The dynamic range was tested and the results met the design specification. 

### 4.3. Anti-Jamming Test and Analysis

**Figure 9:**
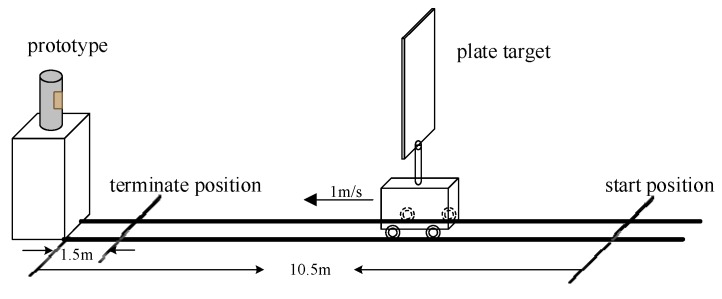
The scenario of a low speed encounter for prototype in the chamber (the metal plate target was fixed on the moving car vertically, and the prototype was placed at one end of the track, loaded by the car moving along the track from 10.5 to 1.5 m at a speed of 1 m/s).

**Figure 10:**
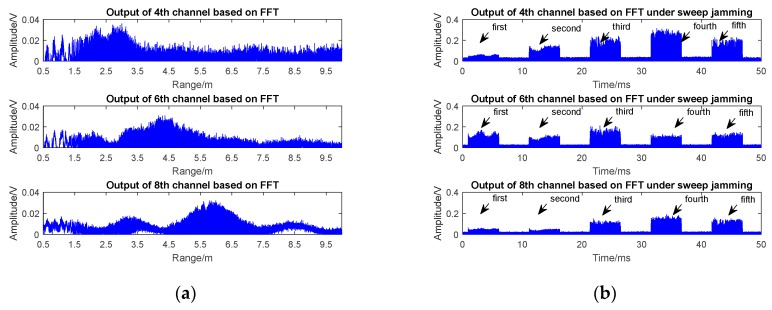
The 4th/6th/8th channel outputs of the prototype. (**a**) Low speed encounter; (**b**) under sweep jamming. (Sweep jamming parameters:$f_{jN}-f_{j0}=100\;\mathrm{MHz}$; $\Delta f_{j}=500\;\mathrm{KHz}$; $T_{dw}=4\;\mathrm{ms}$; AM signal: sine; $f_{jM}=1\;\mathrm{KHz}$.)

The anti-jamming performance of the designed sensor was verified by simulation and jamming experiments. The outputs of the 4th/6th/8th channels of the sensor under real target echo signals in low-speed encounters (shown in Figure 9) are shown in Figure 10a, and the outputs of the 4th/6th/8th channels of the sensor under sweep jamming are shown in Figure 10b.

Figure 10a shows that the 8th harmonic peak at 6 m, the 6th harmonic peak at 4.5 m, the 4th harmonic peak at 3 m, and the peaks of the 8th/6th/4th harmonic signals appear in turn. Considering the practical experiment process where the car began to slow down at a distance of 2 m from the prototype and finally stopped at 1.5 m, the irregular signal at range 1.5–2 m (caused by braking process of the plate target) was normal and did not lead to a false alarm by the sensor. 

The output of the 8th/6th/4th harmonic signals under sweep jamming in Figure 10b was achieved under the condition that the effective jamming signal passed through the BPF’s passband. The update period of the sweeping jammer was 6 ms according to its technical documents. As long as the 4th channel appeared to be jammed, the other two channels were jammed too, as the spectrum of the beat signal is composed of $f_{c}-f_{j0}-k\cdot \Delta f_{j}\pm mf_{m}\pm f_{jM}$. However, the amplitudes of the three channels appeared randomly during one dwell time in the whole sweep jamming cycle. At the first sweep point, the output of the 6th channel was higher than that of the 8th and 4th channels; at the fourth sweep point, the output of the 8th channel was lower than that of the 4th channel, but was higher than the output of the 6th channel; at the second, third, and fifth sweep point, the variation of the output signal amplitude of the three channels satisfied the amplitude change under the target echo, but as the point of sweep frequency changed, the positive edge and negative edge appeared simultaneously. This was essentially different from the outputs of the multi-channels under the real target echo signal and resulted in a mismatch of harmonic timing sequence detection. 

Two thousand simulation experiments using the Monte Carlo method were also conducted. The simulated jamming covered sweep jamming, sweep jamming with AM, and sweep jamming with FM. The AM/FM waveforms consisted of a positive ramp, negative ramp, sin, triangle, square, Gaussian noise, and exponential function. The simulation results showed that the anti-jamming success rate of the designed sensor was 95.5%. With the same jamming parameters, the prototype and the sensor using the structure described in Figure 1 were carried out with the anti-jamming experiment 1000 times, and the success rate of the anti-jamming was 91.3% and 16.7%, respectively. The simulation and anti-jamming experiment results showed that the proposed method improved the anti-sweep jamming capability of the FMCW sensor effectively.

## 5. Conclusions

To overcome the sweep jamming problem faced by the FMCW proximity sensor in a jamming environment, this study designed a short-range FMCW proximity sensor using harmonic timing sequence extraction. Based on an analysis of the failure mechanisms of a proximity sensor under sweep jamming, we proposed an anti-jamming method of multi-channel harmonic timing sequence detection. To consume fewer hardware resources, FFT-based implementation was employed, and a prototype was designed to verify the effectiveness of the method and the improvements in anti-sweep performance. The simulation and experimental results demonstrated that the sensor described in this paper can effectively improve anti-sweep jamming performance under the condition of the same ranging precision $\Delta R=\frac{c}{4\Delta F}$.

## Figures and Tables

**Table 1:** Simulation parameters.

Parameters	Value
Carrier frequency	X-band
FM frequency $f_{m}$/KHz	100
Bandwidth of sensor 2ΔF/MHz	100
Detection range/m	1–15
Target speed/(m/s)	500
Sensor sampling frequency/MHz	5
FFT points	128
SNR/dB	−10

**Table 2:** Implementation complexity analysis (XC3S1000).

	Parameters		
	Number of Slices Flip Flops	Number of 4 input look-up tabels (LUTs)	Number of MULT18X18s
BPF	5256	4573	21
FFT	1502	1281	8
